# Evaluating and Selecting Kinetic and Isotherm Models for Copper and Nickel Removal Using Cow Bone Char as an Adsorbent via Excel Solver Functions

**DOI:** 10.3390/ijms26094316

**Published:** 2025-05-01

**Authors:** Pornmongkol Tansomros, Poramed Aungthitipan, Surachai Wongcharee, Sukanya Hongthong, Torpong Kreetachat, Nopparat Suriyachai, Wipada Dechapanya, Nipada Papukdee, Chatklaw Jareanpon

**Affiliations:** 1 Field of Civil Engineering, Faculty of Engineering, Mahasarakham University, Khamriang, Kantarawichai, Mahasarakham 44150, Thailand; 65010352002@msu.ac.th (P.T.); 65010352001@msu.ac.th (P.A.); 2 Field of Environmental Engineering, Faculty of Engineering, Mahasarakham University, Khamriang, Kantarawichai, Mahasarakham 44150, Thailand; 3 Department of Mechanical Engineering, Faculty of Arts and Science, Chaiyaphum Rajabhat University, Chaiyaphum 36000, Thailand; sukanya.ho@cpru.ac.th; 4 School of Energy and Environment, University of Phayao, Phayao 56000, Thailand; torpong.envi@gmail.com (T.K.); nopparat.su@up.ac.th (N.S.); 5 Faculty of Engineering, Ubon Ratchathani University, Ubonratchathani 34190, Thailand; wipada.d@ubu.ac.th; 6 Department of Applied Statistics, Rajamangala University of Technology Isan, Khon Kaen Campus Mueng Khon Kaen, Khon Kaen 40000, Thailand; nipada.pa@rmuti.ac.th; 7 Department of Computer Science, Faculty of Informatics, Mahasarakham University, Khamriang, Kantarawichai, Mahasarakham 44150, Thailand; chatklaw.j@mus.ac.th

**Keywords:** biochar, cow bone, adsorption, desorption, heavy metal, regeneration

## Abstract

This study explores the effectiveness of cow bone char as a low-cost, eco-friendly, and biodegradable adsorbent for removing Cu(II) and Ni(II) ions from acidic wastewater as challenging is due to heavy metal-contaminated industrial wastewater. Batch adsorption experiments were conducted to evaluate performance, with advanced nonlinear kinetic and isotherm models applied to analyze the adsorption behavior. Model fitting was performed using Microsoft Excel Solver, and model selection was validated using the Akaike Information Criterion and Average Absolute Relative Deviation Percentage. The FL-PFO kinetic model provided the best fit for time-dependent data, while the Liu and Toth isotherm models most accurately described equilibrium adsorption. Maximum adsorption capacities were 110 mg g^−1^ for Cu(II) and 95 mg g^−1^ for Ni(II), with Cu(II) exhibiting faster and more complete removal. Reusability testing over five cycles showed good potential for repeated use, though with gradual efficiency decline due to structural degradation and limited site regeneration. These results confirm the suitability of cow bone char as a sustainable and effective adsorbent for heavy metal removal, particularly in low-resource or decentralized water treatment systems.

## 1. Introduction

**Figure 1:**
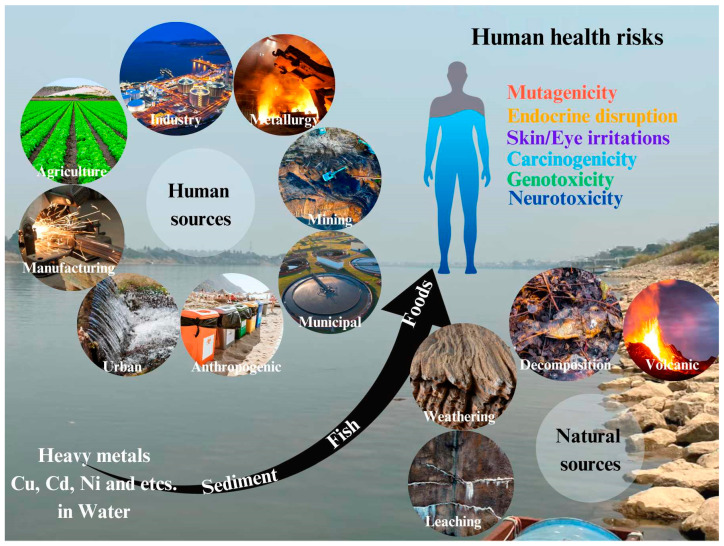
Sources, pathways, and impacts of heavy metal contamination in aquatic ecosystems.

Heavy metals in wastewater pose an extensive environmental and public health risk. These contaminants, consisting of lead, mercury, cadmium, and arsenic, are extremely poisonous, persistent, and non-biodegradable. Upon the implementation into the environment, heavy metals display limited degradation, leading in their accumulation in water, soil, and organisms over extended periods. This accumulation can disrupt aquatic ecosystems by harming aquatic life and destabilizing the natural balance of these systems. Furthermore, the toxic nature of heavy metals poses severe risks to human health, even at low concentrations. Long-term exposure can result in neurological damage, kidney failure, and cancer. Vulnerable populations, such as children and the elderly, are particularly at risk. Additionally, heavy metals can enter the food chain, as contaminated plants and animals accumulate these toxins, which can ultimately affect human consumers. Figure 1 highlights the sources, pathways, and impacts of heavy metal contamination in aquatic ecosystems. These metals originate from both anthropogenic and natural sources. Human activities, such as agricultural runoff (fertilizers and pesticides), industrial discharge [1] (such as copper, cadmium, nickel), mining operations, manufacturing waste, municipal wastewater, and urban runoff, contribute significantly to contamination. Natural sources [2], including rock weathering, soil leaching, volcanic activity, and organic decomposition, also release heavy metals into water bodies. Once in aquatic environments, these metals either bind to sediments or remain suspended in the water, where it accumulates in aquatic organisms like fish.

Consumption of contaminated fish poses a threat to human health. The act of bioaccumulation could lead to genetic mutation, endocrine disruption, carcinogenicity, neurotoxicity, and genotoxicity. These risks signify the need for stronger management and remediation techniques. Furthermore, the presence of heavy metals in wastewater could result in soil degradation, which may negatively affect the agricultural sector by limiting crop growth and productivity [3]. It is, however, necessary to develop effective methods to remove heavy metals from wastewater. Such techniques that avidly need to learn include adsorption, chemical precipitation [4,5], membrane separation [6] and bioremediation [7], these methods still require developments that achieve more effectiveness, cost efficiency, and better eco-friendliness [8,9,10,11].

Adsorption is a widely utilized approach for the removal of heavy metals from aqueous solutions because of its simple, economical, and high removal rates. It was further proof that the choice of adsorbent plays a foundational role on the performance of the adsorption process, as it has a great impact on metal ion removal from wastewater [12,13]. One promising raw material such agricultural and forestry residues are abundantly available worldwide, offering a substantial resource for product derived from these organic materials is biochar, a carbon-rich substance produced through the pyrolysis of biomass. The production process (fast and slow pyrolysis), which involves heating organic matter to high temperatures in the absence of oxygen, creates a stable form of carbon that can be used for multiple purposes, such as soil enhancement, carbon sequestration, and environmental cleanup [14,15,16]. Previous studies have demonstrated the potential of agricultural waste materials as adsorbents for removing heavy metals from aqueous solutions. Materials such as citrus maxima peel, passion fruit shells, and sugarcane bagasse contain carboxylic acid groups that can serve as exchangeable cation and complexation sites for adsorbing metals like copper, cadmium, nickel, and lead [17]. Green coconut shells have also been used as adsorbents for removing toxic metal ions, with the adsorption capacity following the order: Cu^2+^ > Pb^2+^ > Cd^2+^ > Zn^2+^ > Ni^2+^ [18]. A comprehensive review of the literature also indicates that agricultural by-products and modified materials have high capacities for adsorbing heavy metals, including Pb, Cd, Hg, Cu, Ni, Cr, and Cr [19]. In the literature reviewed, cow bone char has grown to be one of the frontrunners as an adsorbent. Earlier studies have shown the efficiency of cow bone char for removing metal ions from contaminated wastewater [20,21]. Cow bone char, being a natural material, has the advantages of being readily available and possessing good adsorption capacities for these metals. However, it has yet to be studied in a mildly acidic environment.

Optimizing the adsorption process requires a precise understanding of the various mechanisms and the adsorption-pollutant interface which facilitates adsorption of the pollutants. This includes kinetics of adsorption (rate at which heavy metal ions are removed from the solution) and adsorption isotherms (how the quantity adsorbed changes with concentration of metal ions in the solution). Mastering these models is important in achieving the process of interest because, that help to determine maximum removal efficiency of contaminants and to design better systems for water and wastewater treatment [22]. By studying these parameters, it is possible to ascertain the conditions under which cow bone char performs best, which is essential for its use in industrial and environmental applications. Moreover, the application of Excel Solver functions is extremely helpful in estimating the parameters of the adsorption kinetics and isotherm models which aids in comprehending the adsorption mechanisms for increased productivity.

There is various other software such as MATLAB, OriginPro, SigmaPlot, and Excel Solver spread sheet-based program, which are used to compute these models in order to apply them to experimental data. While MATLAB, OriginPro and SigmaPlot conduct multiple iterations of nonlinear regression for model parameter estimation, nevertheless these advanced software tools may not have built-in error functions. On the other hand, Excel Solver offers sufficient capabilities for modeling isotherm and kinetic functions and allows users to implement empirical models and error analysis effectively. Moreover, the Akaike Information Criterion (AIC) is well known for estimating the tradeoff between the fit of the model and the complexity of the model which determines the appropriateness of the mode [23]. It allows researchers to integrate the transforming of the model with the estimating of the parameters as a singular process [23]. While, the corrected Akaike Information Criterion is a modified version of AIC, where an additional penalty term is introduced in order to reduce the bias of AIC for small sample sizes. Nevertheless, the AIC and its AIC_corrected_ counterparts performances have not been assessed well enough within the modeling of adsorption systems. The Average Absolute Relative Deviation Percentage (AARD%) is commonly used to measure goodness of fit, which is another alternative metric that can assist with model performance evaluation [23,24,25].

In this work, the potential of cow bone char as an inexpensive and ecological adsorbent for the removal of Cu(II) and Ni(II) ions from water at low pH is presented and studied. The study of adsorption kinetics and isotherms was done by using Excel Solver to fit the parameters of the appropriate models. This addresses a common challenge in regions or institutions with limited access to advanced software in contributing to the field by offering a practical, accessible methodology for adsorption modeling using a tool (Excel Solver) that is widely available but underutilized for complex nonlinear modeling.

Experimental data collected from the batch adsorption experiments were used to determine the adsorption kinetics and isotherms for copper and nickel ion removal using cow bone char as the adsorbent in acidic solution. The challenging is due to heavy metal-contaminated industrial wastewaters, particularly from electroplating, mining, and battery manufacturing processes, often exhibit acidic pH values, frequently ranging between 1.5 and 3.5. Conducting the study at pH 2 allowed us to closely simulate real-world wastewater conditions, thereby enhancing the environmental relevance and applicability of our findings.

The outcomes of this research may contribute to the development of sustainable and economical strategies for heavy metal removal using cow bone char as an adsorbent. Moreover, the performance of AIC, AIC_corrected_ and percent AARD were also explored for comparing model selection.

## 2. Results and Discussions

### 2.1. Effect of Contact Time and Concentration of Cu(II) Ions and Ni(II) Ions Removal onto Cow Bone Char

**Figure 2:**
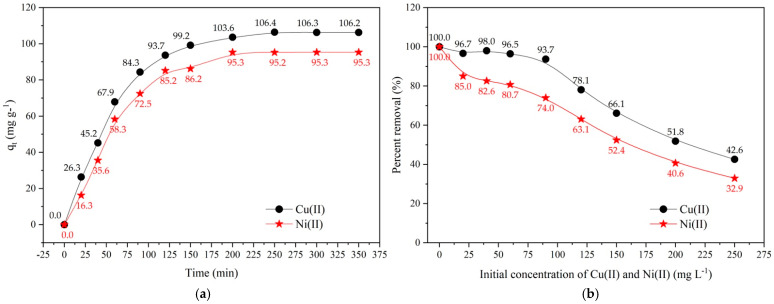
The results of adsorption capacity at time *t* and percent removal; (**a**) effect of contact time and (**b**) effect of concentration.

The rate at which Cu(II) ions and Ni(II) ions are absorbed from synthetic water solutions using cow bone char depends on how long the two types of ions are in contact with each other and how concentration that are at the start as shown in Figure 2a,b. There is a need to establish the best conditions to obtain a high degree of removal at minimum cost. The environment is greatly served by properly solving these problems since cow bone char can be used to improve the treatment of water pollution by removing different heavy metals.

Figure 2a,b show the adsorption behavior of Cu(II) ions and Ni(II) ions by using cow bone char. Figure 2a, the adsorption capacity as a function of contact time reveals a rapid initial uptake for both Cu(II) ions and Ni(II) ions during the first 50 min. This can be attributed to the abundant availability of active adsorption sites on the adsorbent surface. Beyond this point, the adsorption rate slows as these sites become progressively occupied, with equilibrium being achieved at approximately 200 min. At equilibrium, the maximum adsorption capacity for Cu(II) ions is around 110 mg g^−1^, whereas for Ni(II) ions, it stabilizes at approximately 95 mg g^−1^. This suggests that the adsorbent has a higher affinity for Cu(II) ions compared to Ni(II) ions. The overall time-dependent adsorption profile highlights the importance of optimizing contact time for maximum adsorption efficiency in water treatment applications. In terms of Figure 2b examines the percent removal of Cu(II) ions and Ni(II) ions as a function of their initial concentrations. At lower concentrations (0–50 mg L^−1^), the removal efficiency for both ions are nearly 100 percent, indicating effective adsorption due to the availability of sufficient active sites. However, as the concentration increases, the removal efficiency declines, particularly for Ni(II) ions. At higher concentrations, such as 200 mg L^−1^, Cu(II) ions maintain a removal efficiency of approximately 70%, whereas Ni(II) ions drop below 50%. This decrease in removal efficiency is likely due to the saturation of the adsorbent’s active sites at elevated ion concentrations. The consistently higher removal efficiency of Cu(II) ions across all concentrations highlights the material’s greater selectivity and affinity for Cu(II) ions compared to Ni(II) ions. Overall, the figure demonstrates the effective adsorption performance of the material, particularly for Cu(II) ions, making it suitable for applications in water purification and environmental remediation. The findings emphasize the importance of understanding the kinetics of adsorption and the influence of initial ion concentrations to optimize the performance of adsorbent materials in real-world scenarios.

### 2.2. Kinetic Adsorption Studies

The adsorption kinetics of cow bone char for Cu(II) ions and Ni(II) ions were investigated through several kinetic models: Pseudo First Order (PFO), Pseudo Second Order (PSO), Fractal-like Pseudo First Order (FL-PFO), Fractal-like Pseudo Second Order (FL-PSO), Diffusion-Chemisorption (DC) and Elovich. Each model was assessed using adsorption capacity (*q_t_*) and a variety of error metrics, including RMSE, Chi-square, NSD, ARE, SSE, EABS, HYBRID, MPSD and R-square, to evaluate their fit to the experimental data as shown in Table 1.

PFO model predicted *q_t_* values of 108.4 mg g^−1^ and 98.9 mg g^−1^ for copper and nickel, respectively, with *k_1_* values of 0.016 and 0.014 g mg^−1^min^−1^ Although the model achieved high *R*^2^ values of 0.9961 (copper) and 0.9871 (nickel), its higher *RMSE* (2.435 for copper and 4.161 for nickel) and *Chi-square* (*χ*^2^) (1.091 and 2.340, respectively) suggest that the model only moderately fits the data and may struggle to capture the complex dynamics of the adsorption process. PSO model, while improving the predicted *q_t_* values to 131.7 mg g^−1^ (copper) and 123.5 mg g^−1^ (nickel), showed a slight decline in *R*^2^ values (0.9819 for copper and 0.9697 for nickel). Additionally, the model exhibited significantly higher *RMSE* values (5.243 for copper and 6.367 for nickel) and higher *χ*^2^ metrics, indicating a weaker fit compared to the fractal-like models. These results suggest that the PSO model, despite its theoretical basis for chemisorption processes, may not be ideal for accurately modeling this specific system.

On the other hand, the FL-PFO model had better accuracy evidenced by the lower *RMSE* values of 1.339 and 1.969 and the respective *χ*^2^ values of 0.327 and 0.566 for Cu(II) and Ni(II) ions. Since the FL-PFO model had high *R-square* values of 0.9988 for copper and 0.9971 for Ni(II) ions, it indicates that the model is fitted to the data well with kinetic adsorption and the heterogeneity of adsorption sites that need to be filled, which are not addressed in normal models. In the same manner, the FL-PSO model had improved results since the qt values for Cu(II) and Ni(II) ions were at 112.3 and 99.4 mg g^−1^ respectively, with high *R-square* values of 0.9971 for both metals. The error metrics for FL-PSO were in the same range as those of FL-PSO, even so, this goes to show the great applicability of the fractal-like models for adsorption processes in complex systems like cow bone char. The Diffusion-Chemisorption (DC) model had predicted much higher *q_t_* values of 254.6 mg g^−1^ (copper) and 282.8 mg g^−1^ (nickel) due to purported high adsorption capacity. With the model’s performance metrics having lower *R-square* values to 0.9429 and 0.9449 as well as much higher *RMSE* and *χ*^2^ values, this indicates a poor fit to the experimental data. The magnitude of the difference indicates that although the DC model incorporates some elements of the adsorption mechanisms, it might have overestimated the capacity, or the kinetics of the system under study were not represented properly. In a broad sense, the findings suggest that the fractal-like models (FL-PFO and FL-PSO) offer the best fit for cow bone char adsorption kinetics of Cu(II) ions and Ni(II) ions. These models describe the adsorption process which unlike the ideal process, allows for the complexities of bone char adsorption. The more concerning aspect is the degree of overestimation that the DC model presents in terms of its high error metrics. These results illustrate the usefulness of fractal-like models as stronger alternatives for the design and removal of heavy metals for environmental and wastewater treatment processes.

**Figure 3:**
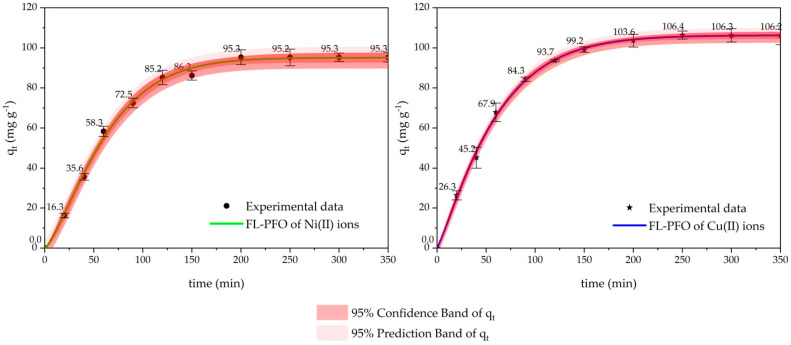
FL-PFO, Experimental data and prediction kinetic adsorption model of copper and nickel removal onto cow bone char.

Figure 3 illustrates the comparison of the adsorption kinetics of Ni(II) and Cu(II) ions on cow bone biochar as predicted by the Fractional Logistic FL-PFO model. The respective plots depict the fitted curves of the model along with the experiment data points for Ni(II) ions and Cu(II) ions. In the graphs, the 95% confidence and prediction bands for the adsorption capacity, *q_t_*, are also presented as functions of time. The model’s estimates for the data align well which shows that the experimental data agrees with the model’s predictions of the adsorption process. These results point out that the adsorption mechanisms for both metal ions are predominantly associated with biochar physical adsorption processes. These findings are particularly significant in advancing the understanding of adsorption kinetics in biochar applications for heavy metal removal, providing insights into the model’s reliability and practical implications for wastewater treatment.

**Figure 4:**
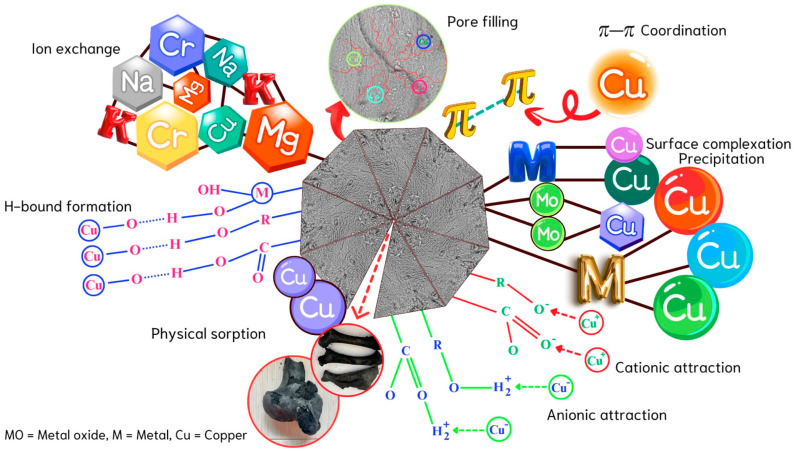
Possible mechanisms involved in the adsorption process of Cu(II) ions onto cow bone char.

**Figure 5:**
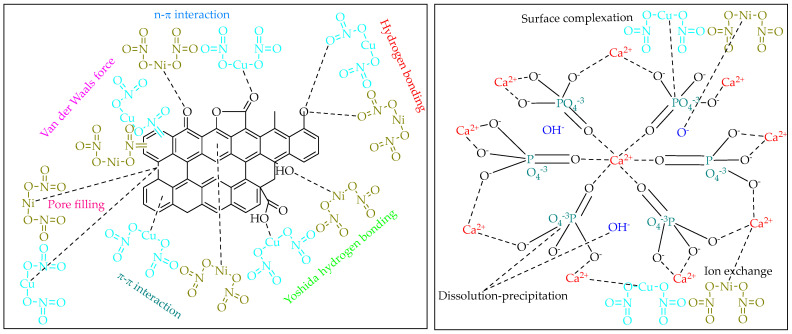
Proposed molecular bonding mechanism for Ni(II) and Cu(II) ions adsorption onto cow bone char surface.

Figure 4 exhibits the putative mechanisms influencing the adsorption of Cu(II) ions onto the surface of cow bone char, while the mechanisms for Ni(II) ions are not included here due to the resemblance to those of copper. This detailed representation elucidates the complex interactions and mechanisms involved in Cu(II) ions binding [26], illustrating essential processes such ion exchange, surface complexation, pore filling, cationic attraction, anionic attraction H-bound formation, π-π coordination and physical adsorption [21,27,28]. However, Figure 5 represents proposed molecular bonding for divalent metal ions adsorption onto cow bone char surface to clarify the adsorption mechanism. In addition, it is also crucial to recognize that the chemical speciation of Cu(II) and Ni(II) ions is highly dependent on the pH of the surrounding environment. As the pH increases, both Cu(II) and Ni(II) ions undergo progressive hydrolysis, leading to the formation of various hydroxy complexes. For Cu(II), these transformations typically result in species such as Cu(OH)^+^, Cu(OH)_2_ (a neutral species), and the negatively charged complex Cu(OH)_4_^2−^. Similarly, Ni(II) ions form hydrolyzed species including Ni(OH)^+^, Ni(OH)_2_, and Ni(OH)_4_^2−^ as the pH shifts toward more alkaline conditions [29]. These changes in speciation significantly influence the ions’ solubility, mobility, and reactivity in aqueous systems.

### 2.3. Adsorption Isotherm Studies

The adsorption behavior of Cu(II) ions and Ni(II) ions onto cow bone char was analyzed using several isotherm models (Table 2), including Langmuir, Freundlich, Temkin, Khan, Liu, and Toth. These models provide valuable insights into the adsorption mechanisms and efficiency. The Langmuir model demonstrated a higher maximum adsorption capacity (*q_max_*) for copper (104.7 mg g^−1^) compared to nickel (92.9 mg g^−1^), indicating that copper ions have a greater tendency to form a uniform monolayer on the adsorbent surface. Additionally, the Langmuir equilibrium constant (*k_l_*) for copper (0.573 L mg^−1^) is significantly higher than that for nickel (0.078 L mg^−1^), suggesting a 1stronger affinity of copper ions for the adsorbent. The R^2^ values (0.9848 for copper and 0.9202 for Nickel) further confirm that the Langmuir model better describes the adsorption process of copper than nickel. However, higher *χ*^2^ and *RMSE* values for nickel indicate a poorer fit for the Langmuir model.

The Freundlich isotherm model, which accounts for surface heterogeneity, revealed that copper has a much higher Freundlich constant (*k_f_* = 46.48 mg g^−1^ (L g^−1^)^n^ compared to Nickel (*k_f_* = 22.72 mg g^−1^ (L g^−1^)^n^, indicating a greater adsorption capacity for copper on heterogeneous sites. However, the adsorption intensity parameter (*n_f_*) was higher for nickel (0.273) than copper (0.182), suggesting slightly better adsorption favorability for nickel. Despite this, the *R*^2^ values for the Freundlich model (0.9430 for copper and 0.9030 for nickel) were lower than those of the Langmuir model, indicating a less precise fit.

The Temkin model provides additional insights by examining the heat of adsorption. The heat parameter (*b_t_*) was slightly higher for nickel (18.45 J mol^−1^) compared to copper (14.51 J mol^−1^), suggesting stronger interactions between the adsorbent and nickel ions. On the other hand, the Khan model, which integrates adsorption capacity and energy, predicted a significantly higher *q_max_* for nickel (178.2 mg g^−1^) than copper (101.6 mg g^−1^). This finding highlights the ability of the Khan model to describe the adsorption capacity of Nickel more effectively. The *R-square* values for the Khan model were also high (0.9912 for copper and 0.9635 for nickel), indicating a strong fit to the experimental data.

The Liu and Toth models, which incorporate characteristics of both Langmuir and Freundlich behaviors, were the statistically best for both metals. For copper and nickel, both models gave remarkably similar *q_max_* for copper of 103.2 and 103.2 mg g^−1^ and for nickel of 79.5 and 80.2 mg g^−1^, with very strong *R-square* values for both metals (>0.99). In addition, these models had the lowest error values for *RMSE*, *χ*^2^, *NSD*, and *SSE*, further demonstrating their correctness. The Liu and Toth models seemed to provide the most dependable representation of the metals because their thoroughly expressed both the uniformity and variability of the adsorption sites. However, among all models, The Liu and Toth models were found to be the most accurate for both metals in all the experiments, proving the numerous kinds of adsorption mechanisms. It can be seen that these findings highlight the effectiveness of cow bone char as a multifunctional water treatment adsorbent that favors copper over nickel in both adsorption affinity and capacity.

**Figure 6:**
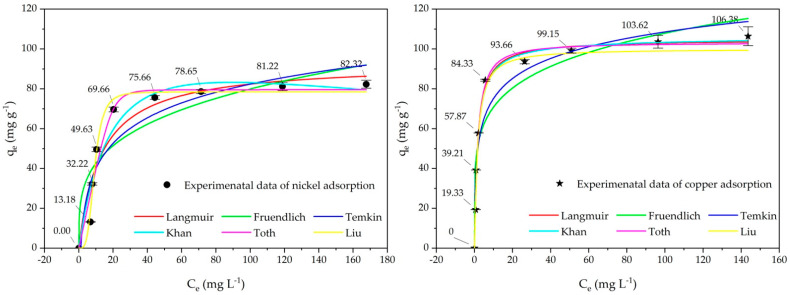
Adsorption isotherms model of copper and nickel removal onto cow bone char adsorbent.

Figure 6 exhibits Cu(II) ions and Ni(II) ions removal adsorption isotherm models on cow bone char adsorbent. The data from the experiments conducted for both nickel and copper were correlated to Langmuir, Freundlich, Temkin, Khan, Toth, and Liu models. The Liu and Toth models developed were evidenced to be more quantitatively accurate for both metals as indicated above due to their versatility in the various types of adsorption isotherms. These suggest the versatility cow bone char possesses as an adsorbent during water treatment processes, where Cu(II) ions is outperformed by Ni(II) ions in regard to adsorption capacity and affinity. As noted in previous studies [21] had been studied thermodynamic adsorption, increasing the temperature enhances the adsorption of Cu(II) and Ni(II) ions onto cow bone char, indicating that the process is endothermic and spontaneous. It can be suggested that this improvement in efficiency at higher temperatures highlights the important role of temperature in optimizing heavy metal removal during the early stages of water treatment. This material supports the elimination of industrial effluent containing Cu(II) ions and Ni(II) ions or divalent metal pollutants and is anticipated to enhance environmental and health protective measures.

### 2.4. Statical Analysis and Akaike’s Information Criterion Its Appropriate Selection

Table 3 provides a detailed comparison of six kinetic adsorption and adsorption isotherm models for the adsorption of Cu(II) ions and Ni(II) ions on cow bon char. The models evaluated include the PFO, PSO, FL-PFO, FL-PSO, DC, and Elovich models. Statistical metrics such as the sum of squared errors (SSE) were used as a key factor of statical data for computing the Akaike’s Information Criterion (AIC) and corrected AIC (AIC_corrected_), which were used to assess the models’ performance.

Among these, the FL-PFO model consistently exhibited the lowest *SSE* values for both Cu(II) ions (16.138) and Ni(II) ions (34.894), indicating superior goodness-of-fit. Furthermore, it achieved the lowest AIC values 10.216 for Cu(II) ions and 18.698 for Ni(II) ions, signifying its ability to balance model accuracy and complexity effectively.

In contrast, the DC and Elovich models showed the highest *SSE* and AIC values, highlighting their inadequacy in describing the adsorption process. The classical PFO and PSO models demonstrated moderate performance, with the PFO model slightly outperforming the PSO model in terms of SSE and AIC for both ions. However, fractional-order models (FL-PFO and FL-PSO) provided substantial improvements, likely due to their ability to account for complex multistep adsorption dynamics. In particular, with the adsorption of Cu(II) ions, the performance of the FL-PFO model was superior to FL-PSO. The effectiveness of the two models was, however, similar when it came to the adsorption of Ni(II) ions. These results indicate that fractional order kinetics are more satisfactory in explaining the adsorption processes, which may include surface activity and diffusion processes. As the results in Table 3 indicate that the FL-PFO model is the most appropriate for estimating the adsorption kinetics of Cu(II) ions and Ni(II) ions onto cow bone char. This highlights the significance of advanced statistical methods for the selection of suitable adsorption kinetic models, for both predictive purpose and practical application, the use of which ensures better model fit. Such information is important for the effective design of adsorption processes for water treatment and removal of heavy metals to make environmental engineering processes more effective.

**Figure 7:**
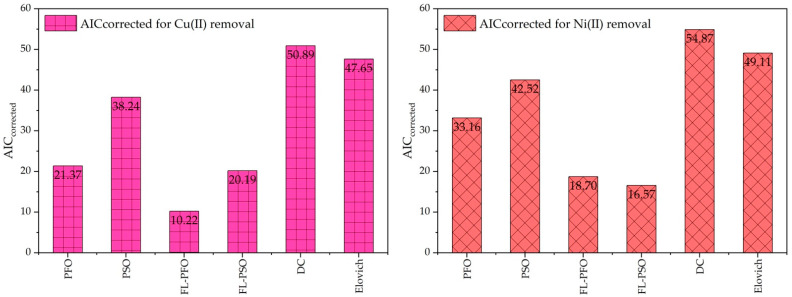
Kinetic adsorption models, AICcorrected for Cu(II) ions and Ni(II) ions removal onto cow bone char.

These visualizations (Figure 7) prove that the most appropriate model for evaluating the adsorption kinetics of Cu(II) ions and Ni(II) ions on cow bone char is the FL-PFO model due to the balance of simplicity and fit of the model to the data. This confirms the results of the analysis in Table 1 in which the FL-PFO model exhibited the highest AICcorrected value in those two cases.

Table 4 evaluates isotherm models such as Langmuir, Freundlich, Temkin, Khan, Toth, and Liu for the adsorption of Cu(II) ions and Ni(II) ions onto cow bone char adsorbent using statistical metrics such as AIC and AICcorrected. These metrics provide a comprehensive assessment of the models’ goodness of fit and predictive accuracy.

For Cu(II) ions adsorption, the Liu model establishes the best performance, with the lowest *SSE* (179.176) and a small AIC_corrected_ value (37.72), indicating its superiority in capturing the adsorption behavior. Although the Langmuir model shows a numerically lower AIC_corrected_ for Cu(II) ions, the Liu model demonstrates superior overall performance across both Cu(II) and Ni(II) ions in terms of multiple error indicators such as lowest *SSE*, strong *R-square* values, and lowest AARD%. The Toth and Khan models also exhibit competitive results, with AIC_corrected_ values of 37.91 and 38.20, respectively, but their slightly higher *SSE* values suggest that the Liu model offers a more precise fit. Conversely, the Freundlich and Temkin models perform poorly, with significantly higher *SSE* values (1227.548 and 701.0, respectively) and the highest AICcorrected values, reflecting their limitations in describing the adsorption process. The Langmuir model, although more accurate than Freundlich and Temkin, remains less effective than the Liu, Toth, and Khan models.

For Ni(II) ions adsorption, the Liu model again outperforms the others, achieving the lowest *SSE* (136.037) and AIC_corrected_ value (35.24). As noted, it is worth mentioning that the Liu model of adsorption and its application is valid for both Cu(II) and Ni(II) ions. Toth model was almost as Cu(II) ions as Khan’s model (46.93) with AIC_corrected_ model coming in seconds at 42.20. The Cu(II) ions adsorption was likewise being modeled by the Freundlich and Temkin models which are the highest in terms of *SSE* and AIC_corrected_ values and, thus, do not work well for Ni(II) ions. The Langmuir model score was better than Liu and Toth, with 44.29 being the AIC_corrected_ score.

**Figure 8:**
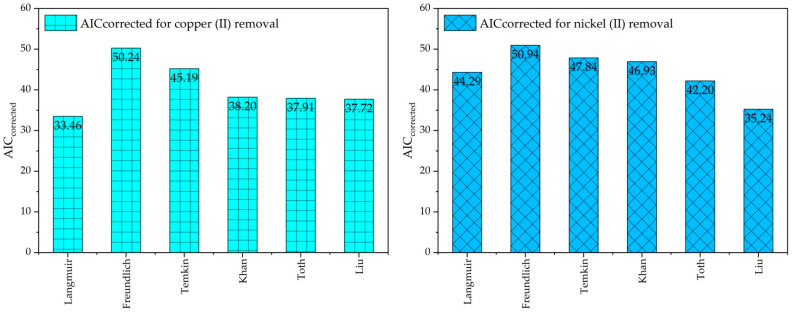
Adsorption isotherm models, AIC_corrected_ for Cu(II) ions and Ni(II) ions removal onto cow bone char.

These results emphasize the efficacy of the Liu model in accurately describing the adsorption isotherms of Cu(II) and Ni(II) ions onto cow bone char. The model proved accurate for both ions, reinforcing its reliability due to the effective, detailed account of the interactions between the adsorbent and the adsorbate, meaning it has potential for further optimization and practical implementations of water treatment. From these findings, it is made clear that the Liu model has strong adaptability and predictive capabilities as seen in Figure 8 which shows the directions of the Cu(II) ions and Ni(II) ions adsorbed onto cow bone char.

**Figure 9:**
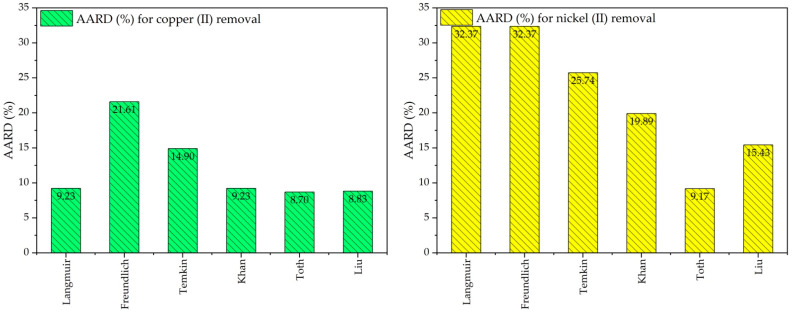
Adsorption isotherm models, AARD (%) for Cu(II) ions and Ni(II) ions removal onto cow bone char.

The bar graphs present in Figure 9, the average absolute relative deviation (AARD) values for six isotherm models Langmuir, Freundlich, Temkin, Khan, Toth, and Liu evaluated for their ability to describe Cu(II) ions and Ni(II) ions adsorption on cow bone char. Lower AARD percent values indicate higher accuracy in fitting the experimental data.

For Cu(II), the Liu and Toth models yield the lowest AARD values (8.83% and 8.70%, respectively), demonstrating their excellent predictive accuracy. The Langmuir and Khan models also perform well, each with an AARD of 9.23%, indicating comparable fitting capability. With an AARD of 14.90%, the Temkin model appears to achieve modest accuracy, whereas the Freundlich model shows the highest AARD of 21.61%, which reflects a poor correlation with the experimental data. When AARD is considered for Ni(II) ions, the Toth model achieves the lowest AARD of 9.17%, followed by the Liu model with 15.43%. The lowest AARD values demonstrate that these models perform better at predicting the results for this metal ion. The Khan model also achieves fairly good results with an AARD of 19.89%. On the contrary, the Langmuir and Freundlich models show the large AARD values of 32.37% which are the unreasonable values obtained from the experiments. The other models show worse performance. The Temkin model with an AARD of 25.74% is ranked in the middle that remains better than the Langmuir and Freundlich models but loses to Toth, Liu and Khan.

The results confirm the assumption that the Toth and Liu models are the most accurate for both Cu(II) ions and Ni(II) ions adsorption, which can be proven with lower AARD rates. The Langmuir and Freundlich models are limited in their ability to predict the value of Ni(II) ions as their AARD values exceed 30%. These findings underscore the robustness of the Toth and Liu models in describing adsorption processes, highlighting their suitability for further applications in modeling metal ion adsorption onto cow bone char.

**Figure 10:**
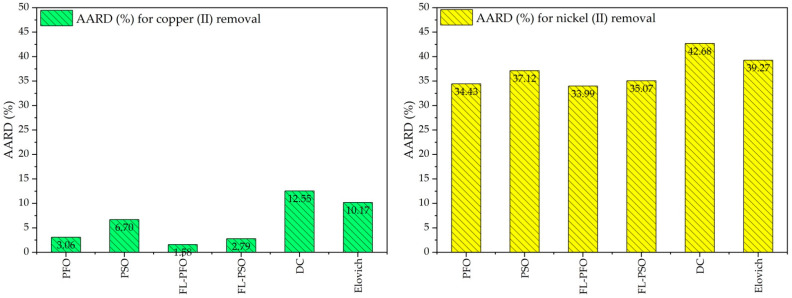
Kinetic adsorption models, AARD (%) for Cu(II) ions and Ni(II) ions removal onto cow bone char.

The graphs display (Figure 10) the Average Absolute Relative Deviation (AARD) percentages for two scenarios: Cu(II) ions removal (left panel, green bars) and Ni(II) ions removal (right panel, yellow bars).

In the Cu(II) ions removal graph, AARD values for different models or processes PFO, PSO, FL-PFO, FL-PSO, DC, and Elovich are presented. The AARD values range from as low as 2.79% for FL-PSO to 12.55% for DC. The FL-PFO model shows the lowest error percentage (1.88%), suggesting it is the most accurate in predicting or modeling copper removal efficiency. On the other hand, the DC and Elovich models have higher AARD values, indicating less reliable performance for this application.

The Ni(II) ions removal graph illustrates significantly higher AARD percentages for the same models. The FL-PFO model again performs better (33.99%), but the overall accuracy of all models is lower compared to copper removal. The DC model has the highest AARD at 42.68%, closely followed by the Elovich model (39.27%), reflecting less precision in modeling or predicting nickel removal outcomes.

The comparison between the two graphs indicates that these models perform better for copper removal than for nickel removal, with consistently lower AARD percentages across all tested methods. This suggests that the processes or dynamics governing copper removal may align better with the assumptions or mechanics of these models.

### 2.5. Desorption and Reusability

**Figure 11:**
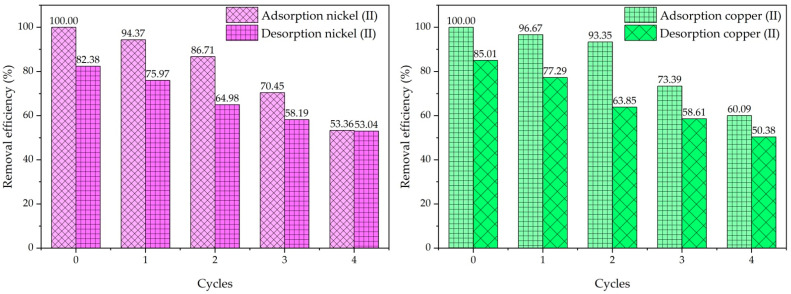
Reusability of cow bone char in removing synthetic Cu(II) ions and Ni(II) ions solutions.

Figure 11, the graphs depict the desorption and reusability performance of the adsorbent material for Ni(II) ions and Cu(II) ions over five consecutive cycles of adsorption and desorption. In the Ni(II) ions removal graph (left), the adsorption efficiency starts at 100% in the first cycle, demonstrating the adsorbent’s high initial capacity. However, with repeated use, the adsorption efficiency progressively declines, dropping to 94.37%, 86.71%, 75.97%, and 70.45% in subsequent cycles. Similarly, the desorption efficiency also decreases, beginning at 82.38% in the first cycle and eventually reducing to 53.04% in the fifth cycle. The declining efficiency suggests a gradual reduction in the adsorbent’s ability to effectively capture and release nickel ions, which could be attributed to saturation of adsorption sites, structural degradation, or reduced binding affinity over time. For Cu(II) ions removal (right graph), a similar trend is observed. The adsorption efficiency starts at 100% in the first cycle and decreases to 96.67%, 93.35%, 85.01%, and 73.39% in subsequent cycles. Desorption efficiency follows a comparable pattern, starting at 85.01% in the first cycle and dropping to 50.38% by the fifth cycle. While copper adsorption appears to retain slightly higher efficiency over repeated cycles compared to nickel, the overall trend of declining performance remains consistent. It confirms this adsorbent demonstrated effective copper and nickel removal with reusability in acidic solutions under laboratory conditions.

These results indicate that the adsorbent material exhibits good reusability in the initial cycles but experiences diminished efficiency with repeated usage. The reduction in performance can be attributed to factors such as incomplete regeneration of adsorption sites during desorption, structural wear of the adsorbent material, or reduced chemical affinity for metal ions after multiple cycles. While the material demonstrates potential for reusability, further optimization may be needed to enhance its durability and maintain consistent adsorption-desorption efficiency over more cycles. It can be concluded that cow bone char is a low-cost, abundantly available material, especially from restaurant waste, making it an economically and environmentally sustainable adsorbent. Its thermal stability and effectiveness across a range of conditions support its potential use in continuous treatment systems.

## 3. Materials and Methods

### 3.1. Synthetic Copper and Nickel Acidic Solution Preparation

To prepare a stock solution with a concentration of 500 mg L^−1^ of acidic synthetic Cu(II), a precise amount of 0.5 g of Cu(II) nitrate (Cu(NO_3_)_2_.3H_2_O, AR grade, Qrec, New Zealand) and Ni(II) nitrate (Ni(NO_3_)_2_.6H_2_O, AR grade, Ajax Finechem) salts were carefully dissolved in 1 L of 2 M hydrochloric acid (HCl). This formulation resulted in a solution with an estimated pH of approximately 2, ensuring the acidic nature of the medium was maintained for subsequent experimental purposes.

Cow bone char was prepared at 400 °C through a process conducted in an oxygen-restricted or oxygen-free environment, utilizing LPG gas as the primary energy source [21]. The heating rate for each production cycle was maintained at approximately 10 °C per minute. After each cycle, the reactor was allowed to cool naturally to ambient temperature of about 25 °C. The resulting cow bone char was finely ground to produce a powder with particle sizes ranging from 0.1 to 0.5 mm. To preserve its physical and chemical properties, the powdered cow bone char was stored in a desiccator. This biochar was subsequently used to assess its adsorption capacity for Cu(II) ions and Ni(II) ions in acidic solution environments.

### 3.2. Kinetic Adsorption Studies

This study examines the adsorption behavior of Cu(II) ions and Ni(II) ions, considering four key variables: contact time, pH (acidic solution less than 2), the quantity of cow bone char, and the concentration of synthetic copper and nickel in an acidic medium with a pH below/or around 2. pH 2 was selected to ensure sufficient solubility of metal ions, minimize precipitation, and evaluate the material’s adsorption capacity under challenging conditions where competition for binding sites is more intense due to protonation effects. A stock solution of copper and nickel with a concentration of 2000 mg L^−1^ was prepared, from which a consistent concentration of 100 mg L^−1^ was utilized across all experimental trials. For the time-dependent study, a copper and nickel solutions at 100 mg L^−1^ was distributed into 11 Erlenmeyer flasks, each containing 1 g of cow bone char. The flasks were agitated and left for various time intervals (0, 10, 20, 30, 40, 60, 90, 120, 150, 200, 250, 300 and 350 min). Following this, the solutions were filtered using a 0.45 GF/C filter to separate the adsorbates (copper and nickel solution). All experiments were conducted in triplicate to ensure reproducibility and reliability of the results.

The concentration of Cu(II) ions and Ni(II) ions adsorbed onto the cow bone char was determined by measuring the solution’s absorbance using an inductively coupled plasma optical emission spectrometer (ICP-OES, Optima 8000, Perkin Elmer Inc., Waltham, MA, USA). The equilibrium adsorption capacity at time *t*, *q_t_* (mg g^−1^) was calculated using Equation (1) for both case studies:

(1) $$q_{t}=\frac{\left(C_{0}-C_{e}\right)\times V}{m}$$

Table 5 shows the adsorption kinetic adsorption models used for evaluating the adsorption capacity and mechanism of adsorption between Cu(II) ions and Ni(II) ions adsorbed onto cow bone char adsorbent.

### 3.3. Adsorption Isotherm Studies

Synthetic copper and nickel solutions with an initial concentration of rage 0–250 mg L^−1^ were meticulously prepared and transferred into Erlenmeyer flasks to facilitate a comprehensive examination of the adsorption isotherm behavior of coper (II) and Ni(II) ions. This investigation aimed to analyze the interaction between the ions and the adsorbent under varying experimental conditions. Copper and nickel concentrations were systematically adjusted to different levels (0, 20, 40, 60, 90, 120, 150, 200, and 250 mg L^−1^). Subsequently, 1 g of cow bone char was added to each flask, and the mixtures (shaking in water bath with 100–120 rpm, at room temperature) were allowed to equilibrate for 24 h. Following this reaction period, the solutions were filtered (0.45 GF/C, Whatman) and stored at temperatures below 4 °C in the refrigerator. The amount of Cu(II) ions and Ni(II) ions adsorbed onto the cow bone char and the absorbance of the filtrates were measured as outlined in Section 2.2. All experiments were performed in triplicate. The equilibrium adsorption capacity was calculated as Equation (2).

(2) $$q_{e}=\frac{\left(C_{0}-C_{e}\right)\times V}{m}$$

Table 6 shows the adsorption isotherm models used for evaluating the adsorption capacity and mechanism of adsorption between Cu(II) ions and Ni(II) ions adsorbed onto cow bone char adsorbent.

### 3.4. Experimental Kinetic and Isotherm Data Fitting in Microsoft Excel

The experimental findings for kinetic and isotherm research studies are often obtained by conducting laboratory tests intended for assessing the adsorption dynamics of copper and nickel removal over various time frames and concentrations, as outlined in Table 7 and Table 8. The kinetic and isotherm data were sourced from a prior investigation employing cow bone char as an adsorbent and acidic copper and nickel solution as the adsorbate. The study utilized batch sorption techniques, following the equations outlined above (Equations (1) and (2)) as obtained from the Section 3.2 and Section 3.3.

Table 7 and Table 8 provide illustrative examples of utilizing the basic Excel Solver function to analyze adsorption data using the Langmuir isotherm model and the PFO kinetic model as following the literature [40,41]. The error function employed in these calculations is specified in the tables as a reference. It is important to note, however, that both tables exclusively focus on the Langmuir and PFO models. For users who intend to apply the Solver function to alternative models, it is necessary to modify the equation in column C of the Excel sheet accordingly. This adjustment should be based on the specific mathematical expressions associated with each model, as detailed in Table 7 and Table 8 of the study. By adapting the equations in column C to reflect the respective model’s formulation, users can leverage the Solver function for accurate parameter estimation across a range of kinetic adsorption and adsorption isotherm models. This approach not only ensures methodological flexibility but also broadens the applicability of the computational framework for diverse adsorption scenarios.

### 3.5. Error Function and Calculation

The experimental findings for kinetic and isotherm research studies were obtained through laboratory tests designed to assess the adsorption dynamics or behavior of Cu(II) ions and Ni(II) ions on cow bone char. Nonlinear regression was applied using Microsoft Excel Solver, which estimates model parameters by minimizing the sum of squared residuals (*SSR*, specifically, Section 3.4) between the experimental data and model predictions. This approach follows the least squares method, a widely accepted technique for parameter optimization in adsorption modeling. By employing nonlinear regression techniques, it was possible to evaluate the interaction between the concentrations of Cu(II) ions and Ni(II) ions and their respective adsorption onto the cow bone char. The error function equations used in this research studies are summarized in Table 9, which summarizes the error functions and statistical equations used to assess the applicability of respective models in the context of wastewater treatment. These statistical metrics provide a robust framework for evaluating the best fitting and reliability of the nominated kinetic and isotherm models in predicting the adsorption behavior under varying experimental conditions.

### 3.6. Statical Analysis and Its Appropriate Selection

This study utilizes Akaike’s Information Criterion (AIC) and the corrected Akaike Information Criterion (AIC_corrected_), based on the guidelines proposed by Akaike in 1998, to assess the performance of adsorption isotherm and kinetic models. In contrast to conventional methods, this approach emphasizes selecting the model with the lowest AIC value, as it strikes a balance between model simplicity and accuracy. The evaluation is conducted using the following formula (Equations (3) and (4)):

(3) $$AIC=N\cdot ln\left(\frac{SSE}{N}\right)+2K$$

(4) $$AIC_{corrected}=AIC+\frac{2K(K+1)}{N-K-1}$$

Here, N is the number of data points, K the model parameters, and *SSE* represents the error sum of squares as indicated in Table 9.

The Average Absolute Relative Deviation percentage (AARD%) is a quantitative measure of the difference between experimental data and theoretical predictions. It conveys the variability between observed and calculated results by means of a percentage; the lower the AARD%, the more accurate the prediction. It is a metric that has more benefits in fields such as kinetics and adsorption isotherms. In these studies, AARD% measures the performance of these models’ PFO, PSO, FL-PFO, FL-PSO, DC, and Elovich equation algorithms while estimating rates of reaction or the rate of adsorption with respect to time. The AARD% helps show which of the adsorption isotherm models, such as Langmuir, Freundlich, Khan, Toth, Liu, and Temkin, indicating a model’s superior fit to the experimental data. Indeed, lower AARD% leads to higher confidence that the model is a good fit to the experimental data. In kinetics and adsorption studies, the most adequate models will have the lowest AARD% values. You can compute AARD% using the following method (Equation (5)):

(5) $$AARD\%=\frac{100}{N}\sum_{i=1}^{N}\left|\frac{q_{\exp,i}-q_{cal,i}}{q_{\exp,i}}\right|$$

In which N represents the total number of experimental data points, *q_exp,i_* is the experimental data and *q_cal,i_* the corresponding value predicted by the model.

### 3.7. Desorption and Reusability

In the study, the reusability of cow bone char was analyzed where Cu(II) and Ni(II) ions were used in an adsorption—desorption cycle. At first, the samples were meticulously rinsed two times with distilled water to eliminate unabsorbed contaminants and ensure that there are no unwanted heavy metals. This was followed by gentle sonication at room temperature for five minutes in a mild 0.1 M HCl (AR grade, Ajax Finechem) solution. In the subsequent, Cu(II) and Ni(II) ions were desorbed from the adsorbent beads during the sonication step, and these ions were released into the HCl supernatant solution. Finally, the supernatant was analyzed using ICP-OES to determine how much of the Cu(II) and Ni(II) ions remained bound to the beads after each desorption step. The above stated steps were repeated along with ICP-OES analysis until the beads proved capable of being reused. After each analysis, the cow bone char which had adsorbed the ions was treated with deionized water to neutralize and remove the remaining acid along with other contaminants. The cleaned adsorbent was then dried to prepare it for subsequent adsorption cycles. Each of the char materials went through these procedures five times in order to evaluate how effective the applied cow bone char.

## 4. Conclusions

This research establishes the potential of cow bone char as a cost-effective and environmentally sustainable adsorbent for the removal of heavy metals, specifically Cu(II) ions and Ni(II) ions, from acidic solutions. The experimental results revealed that cow bone char demonstrates a higher adsorption capacity and affinity for Cu(II) ions compared to Ni(II) ions, attributable to differences in the ions’ interaction with the adsorbent’s surface properties, such as porosity and functional groups. Comprehensive kinetic modeling identified the FL-PFO models as the most accurate in describing the adsorption process. These models effectively accounted for the complex, multistep nature of the adsorption mechanism, including surface heterogeneity and pore diffusion. Among the isotherm models evaluated, the Liu and Toth models provided the best fit, capturing the combined effects of homogeneous and heterogeneous adsorption behaviors. Although classical kinetic and isotherm models provided valuable insight into the adsorption behavior and showed good agreement with the experimental data, we acknowledge that more advanced modeling approaches could offer deeper mechanistic understanding.

Through the effectiveness assessments of multiple adsorption-desorption cycles, it was revealed that cow bone chars have good performance, however, the declining efficiency is attributed to incomplete regeneration, reduced active site availability, and structural degradation. This highlights the need for optimization strategies, such as improved regeneration protocols or chemical modifications, in order to enhance the materials durability for long term applications.

In summary, cow bone char stands out as a feasible compound for the removal of heavy metals in wastewater treatment considering its cost-effectiveness, efficiency and environmental friendliness. It is especially beneficial for the treatment of industrial wastewater associated with divalent metal ions like copper and nickel. This research represents one of the fundamental steps towards novel and environmentally friendly water remediation technology that will lead to improved environmental health and safety.

## Figures and Tables

**Table 1:** Adsorption kinetics model of cow bone char.

Kinetic Adsorption Model of Factor and Error Function	Cu(II) Ions	Ni(II) Ions	Kinetic Adsorption Model of Factor and Error Function	Cu(II) Ions	Ni(II) Ions
Pseudo First Order (PFO)			Pseudo second order (PSO)		
*q_e_* (mg g^−1^)	108.4	98.91	*q_e_* (mg g^−1^)	131.7	123.5
*k_1_* (min^−1^)	0.016	0.014	*k_2_* (g mg^−1^ min^−1^)	0.0001	0.0001
*RMSE*	2.435	4.161	*RMSE*	5.243	6.367
*Chi-square*	1.091	2.340	*Chi-square*	4.494	10.64
*NSD*	230.9	394.7	*NSD*	497.4	604.0
*ARE*	−1.511	−5.132	*ARE*	−2.953	−6.946
*SSE*	53.35	155.83	*SSE*	247.4	364.8
*EABS*	18.48	32.22	*EABS*	44.12	52.94
*HYBRID*	−1.888	−6.647	*HYBRID*	−3.692	−8.683
*MPSD*	10.68	37.60	*MPSD*	20.88	49.12
*R-square*	0.9961	0.9871	*R-square*	0.9819	0.9697
Fractal-like pseudo first order (FL-PFO)			Fractal-like pseudo second order (FL-PSO)		
*q_e_* (mg g^−1^)	106.3	95.1	*q_e_* (mg g^−1^)	112.3	99.4
*k_1_* (g mg^−1^ min^−1^)	0.008	0.004	*k_2_* (g mg^−1^ min^−1^)	0.000017	0.000005
*α*	1.162	1.334	*α*	1.629	1.910
*RMSE*	1.339	1.969	*RMSE*	2.107	1.958
*Chi-square*	0.327	0.566	*Chi-square*	0.973	0.738
*NSD*	127.0	186.8	*NSD*	199.9	185.7
*ARE*	0.103	−0.669	*ARE*	0.672	0.841
*SSE*	16.14	34.89	*SSE*	39.96	34.49
*EABS*	9.40	14.21	*EABS*	15.51	17.19
*HYBRID*	0.128	−0.836	*HYBRID*	0.840	1.051
*MPSD*	0.726	4.728	*MPSD*	4.752	5.946
*R-square*	0.9988	0.9971	*R-square*	0.9978	0.9971
Diffusion-Chemisorption (DC)			Elovich		
*q_e_* (mg g^−1^)	254.6	282.8	*α_e_* (mg g^−1^ min^−1^)	3.526	2.395
*k_DC_* (mg g^−1^ min^−n^)	11.40	8.98	*β_e_* (g mg^−1^)	0.031	0.031
*RMSE*	9.32	10.19	*RMSE*	8.040	8.591
*Chi-square*	16.73	30.91	*Chi-square*	10.10	17.50
*NSD*	883.9	966.8	*NSD*	762.8	815.0
*ARE*	−6.11	−11.98	*ARE*	−3.873	−8.008
*SSE*	781.3	934.7	*SSE*	581.8	664.3
*EABS*	77.19	82.82	*EABS*	68.73	73.19
*HYBRID*	−7.64	−14.98	*HYBRID*	−4.84	−10.01
*MPSD*	43.23	84.73	*MPSD*	27.39	56.63
*R-square*	0.9429	0.9225	*R-square*	0.9575	0.9449

**Table 2:** Results of the isotherm parameters and statistical analysis of cow bone char.

Adsorption Isotherm Model/Factor and Error Function	Cu(II) Ions	Ni(II) Ions	Adsorption Isotherm Model/Factor and Error Function	Cu(II) Ions	Ni(II) Ions
Langmuir			Freundlich		
*q_m_* (mg g^−1^)	104.7	92.9	*k_f_* (mg g^−1^)(L g^−1^)^n^	46.48	22.72
*k_l_* (L mg^−1^)	0.573	0.078	*n_f_*	0.182	0.273
*RMSE*	0.243	0.695	*RMSE*	0.543	0.445
*Chi-square*	5.14	16.34	*Chi-square*	22.72	28.89
*NSD*	487.6	889.8	*NSD*	1238	1287
*ARE*	−4.53	−17.73	*ARE*	−14.75	−23.98
*SSE*	190.2	633.5	*SSE*	1227.5	1327.1
*EABS*	31.15	53.02	*EABS*	79.55	86.98
*HYBRID*	6.57	32.14	*HYBRID*	37.42	58.88
*MPSD*	0.288	2.186	*MPSD*	1.627	3.837
*R-square*	0.9848	0.9202	*R-square*	0.9430	0.9030
Temkin			Khan		
*b_t_* (J mol^−1^)	14.51	18.45	*k_k_* (L mg^−1^)	0.603	0.033
*k_t_* (L mol^−1^)	17.749	0.868	*a_k_*	0.992	1.342
*RMSE*	0.000059	0.000027	*q_m_* (mg g^−1^)	101.6	178.2
*Chi-square*	13.62	21.61	*RMSE*	0.268	0.664
*NSD*	936.0	1084.4	*Chi-square*	5.17	13.61
*ARE*	−8.56	−16.98	*NSD*	486.0	789.6
*SSE*	700.8	941.0	*ARE*	−4.73	−15.62
*EABS*	56.78	73.13	*SSE*	189.0	498.8
*HYBRID*	19.24	37.49	*EABS*	30.26	47.06
*MPSD*	0.797	2.345	*HYBRID*	6.73	25.85
*R-square*	0.9674	0.9312	*MPSD*	0.300	1.766
			*R-square*	0.9912	0.9635
Toth			Liu		
*q_m_* (mg g^−1^)	103.2	78.5	*q_m_* (mg g^−1^)	103.2	80.2
*kth* (L mg^−1^)	0.502	0.109	*k_g_* (L mg^−1^)	0.601	0.869
*nth*	1.146	0.262	*n_g_*	0.894	0.739
*RMSE*	0.128	0.185	*RMSE*	0.087	0.080
*Chi-square*	4.986	3.510	*Chi-square*	4.915	5.492
*NSD*	478.3	383.8	*NSD*	473.3	412.4
*ARE*	−3.614	−4.540	*ARE*	−3.289	−5.116
*SSE*	183.0	117.9	*SSE*	179.2	136.0
*EABS*	31.17	26.69	*EABS*	31.13	23.94
*HYBRID*	5.798	4.293	*HYBRID*	5.521	6.558
*MPSD*	0.239	0.245	*MPSD*	0.222	0.400
*R-square*	0.9915	0.9913	*R-square*	0.9916	0.9901

**Table 3:** Akaike’s information criterion kinetic models of Cu(II) ionsand Ni(II) ions removal onto cow bon char adsorpbent.

Model	Cu(II) Ions Kinetic Adsorption Model					Ni(II) Ions Kinetic Adsorption Model				
	N	K	SSE	AIC	AIC_corrected_	N	K	SSE	AIC	AIC_corrected_
PFO	11	2	53.345	21.367	22.867	11	2	155.825	33.159	34.659
PSO	11	2	247.357	38.242	39.742	11	2	364.843	42.517	44.017
FL-PFO	11	3	16.138	10.216	13.644	11	3	34.894	18.698	22.127
FL-PSO	11	3	39.965	20.191	23.619	11	2	34.495	16.572	18.072
DC	11	2	781.276	50.893	52.393	11	3	934.708	54.865	58.294
Elovich	11	2	581.819	47.651	49.151	11	2	664.297	49.109	50.609

**Table 4:** Akaike’s Information Criterion isotherm models of Cu(II) ionsand Ni(II) ions removal onto cow bon char adsorpbent.

Model	Cu(II) Ions Adsorption Isotherm Model					Ni(II) Ions Adsorption Isotherm Model				
	N	K	SSE	AIC	AIC_corrected_	N	K	SSE	AIC	AIC_corrected_
Langmuir	9	2	190.173	31.46	33.46	9	2	633.454	42.29	44.29
Freundlich	9	2	1227.548	48.24	50.24	9	2	1327.07	48.94	50.94
Temkin	9	2	7.01 × 10^2^	43.19	45.19	9	2	9.41 × 10^2^	45.84	47.84
Khan	9	3	188.961	33.40	38.20	9	3	498.793	42.13	46.93
Toth	9	3	182.989	33.11	37.91	9	3	294.658	37.40	42.20
Liu	9	3	179.176	32.92	37.72	9	3	136.037	30.44	35.24

**Table 5:** Kinetic adsorption models used for wastewater treatment.

Named of the Model	Model Equation	Reference
Pseudo first order (PFO)	$q_{t}{=q}_{e}\cdot \left[1-\exp(-k_{1}\cdot t)\right]$	[30]
pseudo second order (PSO)	${\;q}_{t}=\frac{k_{2}{{\cdot q}_{e}}^{2}\cdot t}{{1+q}_{e}{\cdot k}_{2}\cdot t}$	[30]
Fractal like-pseudo first order (FL-PFO)	${\;q}_{t}{=q}_{e}\cdot \left(1-\exp\left(-k_{1}{\cdot t}^{\alpha }\right)\right)$	[31]
Fractal like-pseudo second order (FL-PSO)	$q_{t}=\frac{k_{2}{{\cdot q}_{e}}^{2}{\cdot t}^{\alpha }}{{1+k}_{2}{\cdot q}_{e}{\cdot t}^{\alpha }}\;$	[31]
Diffusion-Chemisorption (DC)	$q_{t}=\frac{q_{e}{\cdot k}_{\mathrm{DC}}{\cdot t}^{1/2}}{k_{\mathrm{DC}}{\cdot t}^{1/2}{+q}_{e}}$	[32]
Elovich	$q_{t}=\frac{1}{\beta }\cdot \ln(1+\alpha \cdot \beta \cdot t)$	[33]

**Table 6:** Adsorption isotherm models used for wastewater treatment.

Named of the Model	Model Equation	Reference
Langmuir	$q_{e}{=q}_{m}\cdot k_{l}\frac{C_{e}}{{1+k}_{l}\cdot C_{e}}$	[34]
Freundlich	$q_{e}{=k}_{f}\cdot C_{e}^{{1/n}_{f}}$	[35]
Temkin	$q_{e}{=b}_{t}\cdot {\ln(k}_{t}\cdot C_{e})$	[36]
Khan	$q_{e}=\frac{q_{m}\cdot k_{k}\cdot C_{e}}{{{(1+k}_{k}\cdot C_{e})}^{a_{k}}}$	[37]
Liu	$q_{e}=\frac{q_{m}\cdot {{(k}_{g}\cdot C_{e})}^{{1/n}_{g}}}{{1+(k}_{g}\cdot C_{e}{)}^{{1/n}_{g}}}$	[38]
Toth	$q_{e}{=q}_{m}\frac{k_{\mathrm{th}}\cdot C_{e}}{{{(1+(k}_{\mathrm{th}}\cdot C_{e}{)}^{n_{\mathrm{th}}})}^{{1/n}_{\mathrm{th}}}}$	[39]

**Table 7:** Example solution of Microsoft Excel solver functions for Langmuir isotherm model.

Row/ Column	A, *C_e_*	B, *q_e, exp_*	C, *q_e, cal_*	D, Upper CI	E, Lower CI	F, Residual	G, Residaul^2^	H, Langmuir Isotherm Model Factors		I, Error Function Statistical Results	
1	0	0	0.00	12.33	−12.33	0.00	0.00	*kl*	0.573	*RMSE*	0.243
2	0.67	19.33	29.07	41.39	16.74	−9.74	94.83	*q_m_*	104.736	*Chi-square*	5.148
3	0.79	39.21	32.65	44.98	20.33	6.56	43.02	*SSR*	190.173	*NSD*	487.561
4	2.13	57.87	57.58	69.91	45.26	0.29	0.08	Mean of *q_e,exp_*	67.061	*ARE*	−4.529
5	5.67	84.33	80.10	92.42	67.77	4.23	17.91	df	7.000	*SSE*	190.173
6	26.34	93.66	98.23	110.56	85.91	−4.57	20.91	SE of *q_e,exp_*	5.212	*EABS*	31.144
7	50.85	99.15	101.26	113.59	88.94	−2.11	4.47	Critical t	2.364	*HYBRID*	6.569
8	96.38	103.62	102.88	115.2	90.55	0.74	0.55	CI	12.325	*MPSD*	0.287
9	143.62	106.38	103.48	115.81	91.16	2.90	8.41			*R-square*	0.9848

**Noted**: *q_e, cal_* = y_fit = *q_e_* = q_m_ × (kl × (C_e_/(1 + kl × C_e_))) = column C; *SSR* = SUM(G1:G9); Mean of *q_e,exp_* = AVERAGE(B1:B9); df = COUNT(B1:B9) − COUNT(H1:H2); SE of *q_e,exp_* = SQRT(SUM((B1:B9 − C1:C9)^2^)/H5); R-square = 1 − (SUM((B1:B9 − C1:C9)^2^)/(SUM((B1:B9-H4)^2^))); Critical t = TINV(0.05,H5); CI = H6 × H7; *SSE* = SUM((C1:C9 − B1:B9)^2^); *Chi-square* = SUM(((C2:C9 − B2:B9)^2^)/(B2:B9)); *ARE* = (100/(COUNT(B2:B9))) × (SUM((B2:B9 − C2:C9)/(B2:B9))); *RMSE* = SQRT(SUM((B1:B9 − C1:C9)^2^)/(COUNT(C1:C9) − 2)); *HYBRID* = (100/((COUNT(B2:B9)) − (COUNT(H1:H2)))) × (SUM((B2:B9 − C2:C9)/(B2:B9))); *MPSD* = 100 × SQRT((1/COUNT(B2:B9 − H1:H2) × (SUM((B2:B9 − C2:C9)/(B2:B9))^2^))).

**Table 8:** Example solution of Microsoft Excel solver functions for PFO kinetic model.

Row/ Column	A, Time, *t*	B, *q_t, exp_*	C, *q_t, cal_*	D, Upper CI	E, Lower CI	F, Residual	G, Residaul^2^	H, PFO Model Factors		I, Error Function Statistical Results	
1	0	0.00	0.00	0.89	−0.89	0.00	0.00	*k_1_*	0.016	*RMSE*	0.24
2	20	26.33	29.06	29.95	28.17	2.73	7.46	*q_e_*	108.411	*Chi-square*	1.091
3	40	45.21	50.33	51.22	49.45	5.12	26.24	*SSR*	190.17	*NSD*	230.965
4	60	67.87	65.90	66.79	65.01	−1.97	3.87	Mean of *q_t,exp_*	76.277	*ARE*	−1.511
5	90	84.33	81.79	82.68	80.91	−2.54	6.44	df	9.00	*SSE*	53.345
6	120	93.66	91.74	92.63	90.86	−1.92	3.68	SE of *q_t,exp_*	0.392	*EABS*	18.479
7	150	99.15	97.97	98.86	97.09	−1.18	1.38	Critical t	2.262	*HYBRID*	−1.888
8	200	103.62	103.63	104.51	102.74	0.01	0.00	CI	0.887	*MPSD*	10.682
9	250	106.38	106.22	107.11	105.33	−0.16	0.03			*R-square*	0.9961
10	300	106.26	107.41	108.29	106.52	1.15	1.31				
11	350	106.24	107.95	108.84	107.06	1.71	2.93				

**Noted**: *q_t, cal_* = y_fit = *q_t_* = q_e_ × (1 − EXP(− k1 × t)) = column C; *SSR* = SUM(G1:G11); Mean of *q_e,exp_* = AVERAGE(B1:B11); df = COUNT(B1:B11) − COUNT(H1:H2); SE of *q_e,exp_* = SQRT(SUM((B1:B11 − C1:C11)^2^)/H5); R-square = 1 − (SUM((B1:B11 − C1:C11)^2^)/(SUM((B1:B11-H4)^2^))); Critical t = TINV(0.05,H5); CI = H6 × H7; *SSE* = SUM((C1:C11 − B1:B11)^2^); *Chi-square* = SUM(((C2:C11 − B2:B11)^2^)/(B2:B11)); *ARE* = (100/(COUNT(B2:B11))) × (SUM((B2:B11 − C2:C11)/(B2:B11))); *RMSE* = SQRT(SUM((B1:B11 − C1:C11)^2^)/(COUNT(C1:C11) − 2)); *HYBRID* = (100/((COUNT(B2:B11)) − (COUNT(H1:H2)))) × (SUM((B2:B11 − C2:C11)/(B2:B11))); *MPSD* = 100 × SQRT((1/COUNT(B2:B11 − H1:H2) × (SUM((B2:B11 − C2:C11)/(B2:B11))^2^))).

**Table 9:** Error function or statistical analysis equation used for selecting the best fitting of kinetic adsorption and adsorption isotherm models.

Named of Error Function	Error Function Equation	Reference
*SSE*	$\overset{n}{\underset{i=1}{\sum }}{{(q}_{e,\;\exp}{\;–\;q}_{e,\;\mathrm{cal}})}^{2}\;$	[42]
*Chi-square*	$\overset{n}{\underset{i=1}{\sum }}\frac{{{(q}_{e,\;\mathrm{cal}}-\;q_{e,\;\exp})}^{2}}{q_{e,\;\exp}}$	[43]
R-square	$1\;-\frac{\sum_{n=1}^{n}{{(q}_{e,\;\exp}{\;–\;q}_{e,\;\mathrm{cal}})}^{2}}{\sum_{n=1}^{n}{{(q}_{e,\;\exp\;}–\;\bar{{\;q}_{e,\;\exp}})}^{2}}$	[44]
*ARE*	$\frac{100}{n}\cdot \overset{n}{\underset{i=1}{\sum }}\left(\frac{{(q}_{e,\;\exp}{\;–\;q}_{e,\;\mathrm{cal}})}{q_{e,\;\exp}}\right)$	[43]
*RMSE*	$\sqrt{\frac{\sum_{n=1}^{n}{{(q}_{e,\;\exp}{\;–\;q}_{e,\;\mathrm{cal}})}^{2}}{n-p}}$	[45,46,47]
*HYBRID*	$\frac{100}{n-p}\cdot \overset{n}{\underset{i=1}{\sum }}\left(\frac{{(q}_{e,\;\exp}{\;–\;q}_{e,\;\mathrm{cal}})}{q_{e,\;\exp}}\right)$	[48]
*MPSD*	$100\cdot \sqrt{\frac{1}{n-p}\cdot \overset{n}{\underset{i=1}{\sum }}{\left(\frac{{(q}_{e,\;\exp}{\;–\;q}_{e,\;\mathrm{cal}})}{q_{e,\;\exp}}\right)}^{2}}$	[49]
*NSD*	$100\cdot \sqrt{\frac{1}{n-1}\cdot \overset{n}{\underset{i=1}{\sum }}{\left(\frac{{(q}_{e,\;\exp}{\;–\;q}_{e,\;\mathrm{cal}})}{q_{e,\;\exp}}\right)}^{2}}$	[50,51]
*EABS*	$\overset{n}{\underset{i=1}{\sum }}{\left|{(q}_{e,\;\mathrm{cal}}-\;{\;q}_{e,\;\exp})\right|}_{i}$	[52]

## Data Availability

The data that support the findings of this study are available from the corresponding author upon reasonable request.
